# *Corynebacterium phoceense* – a rare *Corynebacterium* species isolated from a urine sample

**DOI:** 10.1099/acmi.0.000197

**Published:** 2021-01-27

**Authors:** Claudia M. Barberis, Germán M. Traglia, Marisa N. Almuzara, Danilo J. P. G. Rocha, Carolina S. Santos, Eric R. G. R. Aguiar, Luis G. C. Pacheco, Carlos A. Vay

**Affiliations:** ^1^​ Universidad de Buenos Aires, Facultad de Farmacia y Bioquímica, Departamento de Bioquímica Clínica, Hospital de Clínicas José de San Martín, Ciudad Autónoma de Buenos Aires, Argentina; ^2^​ Instituto de Ciencias da Saúde, Universidade Federal da Bahia, Salvador-BA, Brasil

**Keywords:** *Corynebacterium*, MALDI-TOF MS, urinary tract infection

## Abstract

*
Corynebacterium* spp. are Gram-positive rods that are recognized to cause opportunistic diseases under certain predisposing clinical conditions. Some species have been described in urinary tract infections. In this report we document a new episode of urinary tract infection caused by *Corynebacterium phoceense* and describe the whole-genome sequencing, phenotypic characteristics and mass spectra obtained by matrix-assisted desorption/ionization time-of-flight mass spectrometry (MALDI-TOF MS). Based on genome identification and DNA-to-DNA hybridization, we can assume that our strain is the second isolate of *C. phoceense* to be described in a urine sample. No other infectious diseases have been reported to be associated with this species.

## Introduction

*Corynebacterium* spp. are Gram-positive irregular rod micro-organisms that are recognized to cause opportunistic diseases under certain predisposing clinical conditions [1]. Some species have been described in urinary tract infections (UTIs). The most common urinary pathogens include *Corynebacterium urealyticum* and *Corynebacterium riegelii* [2, 3]. The role that *C. urealyticum* plays in UTIs, primarily in association with alkali-encrusted cystitis, has been clearly established. *C. riegelii,* which was first isolated from females with UTIs, has been reported in fatal cases of urosepsis [4, 5]. In recent years, there has been an interest in rare or infrequently described *Corynebacterium* species, which are emerging opportunistic pathogens, such as *Corynebacterium glucuronolyticum *, *Corynebacterium coyleae* and *Corynebacterium aurimucosum,* which have been recovered from patients with UTIs, and are considered to be potential uropathogens [6–8]. Recently a new species, *Corynebacterium phoceense*, has been isolated from the urine of a kidney transplant recipient [9]. We highlight the importance of identifying coryneform bacteria to the species level whenever they are recovered in pure culture from clinical specimens, since they can be the cause of significant infections.

In recent decades, the taxonomy of this genus has changed to a large extent, with many new species now associated with human diseases. Taxonogenomics is a new approach that includes whole-genome based analysis as a tool that is complementary to phenotypic identification and matrix-assisted desorption/ionization time-of-flight mass spectrometry (MALDI-TOF MS) in the description of new bacterial species [10].

In this report we document a new episode of UTI caused by *C. phoceense* and describe the whole-genome sequencing, phenotypic characteristics and mass spectra obtained by MALDI-TOF MS.

## Methods

The strain was isolated from a urine sample obtained from a 47-year-old woman who experienced lower urinary tract symptoms (dysuria and incontinence).

The patient reported undergoing biureteral reimplantation surgery during childhood due to a congenital malformation. The patient had not received antibiotic therapy prior to urine sample collection. The urine sample was cultured aerobically on cystine**–**lactose**–**electrolyte-deficient (CLDE) agar (Britania, Buenos Aires, Argentina) at 35 °C and on sheep blood agar (bioMérieux, Marcy l’Etoile, France) at 35 **°**C in a 5 % CO_2_ atmosphere.

The phenotypic profile was obtained in duplicate using conventional biochemical tests – type of metabolism, lipophilicity, nitrate reduction, aesculin hydrolysis, Christie–Atkins–Munch–Peterson (CAMP) test, and acid production from glucose, maltose, sucrose, lactose and ribose according to the scheme proposed by Funke *et al*. [1] – and using API Coryne systems according to the manufacturer’s specifications (bioMérieux, Marcy l´Etolile, France).

Antimicrobial susceptibility was determined using the epsilometer test (*E*-test) (bioMerieux) on Mueller–Hinton agar supplemented with 5 % sheep blood and the inoculum was equivalent to the 0.5 McFarland standard. Plates were incubated aerobically at 37 °C for 24 h [11]. The interpretative categories for the MICs obtained were used following the Clinical and Laboratory Standards Institute (CLSI), M45 [12].

The isolate was also identified by MALDI-TOF MS. With this technology the ribosomal proteins liberated from bacteria were ionized and detected by mass spectrometry (MS). Mass spectra were generated and the data were analysed with regard to spectrum peak frequency, position and intensity. These spectra were then compared to entries found in a database, giving rise to a degree of match in order to provide identification to the genus and species level [13].

Mass spectra were acquired using the MALDI-TOF MS spectrometer in a linear positive mode (Microflex, Bruker Daltonics) in an *m*/*z* range of 2000 to 20 000 using a Microflex LT controlled by FlexControl version 3.4 software (Bruker).

The genomic DNA of our isolate strain was extracted and purified using the NucleoSpin Microbial DNA kit (Macherey-Nagel, Germany) according to the manufacturer’s guidelines. The concentration and purity (A_260_/A_280_ ratio=1.80) of genomic DNA was determined by spectrophotometry.

Whole-genome sequencing was performed using the Ion Torrent S5 platform with the Ion 540 Chip for 200 bp libraries (Thermo Fisher Scientific). From starting genomic DNA concentrated at 17.3 ng/µl, 100 ng was taken for enzymatic DNA fragmentation using the Ion Xpress Plus Fragment Library kit, according to the manufacturer’s protocol. A specific barcode from the Ion Xpress Barcode Adapters kit was then ligated to the fragments. The EGel SizeSelect Agarose Gel kit was used for the selection of ~200 bp fragments. All sample purification steps were performed using the ProNex Size-Selective Purification System kit (Promega) at a ratio of 2 : 1. Equalization of the library was performed with the Ion Library Equalizer kit, resulting in a final concentration of ~100 pM. The preparation of templates for sequencing strictly followed the protocols of the Ion 540 Kit – OT2, Ion 540 Chip, Ion OneTouch and Ion OneTouch ES instruments. When required, DNA concentrations were estimated using Qubit High Sensitivity Assay kits.

*De novo* assembly was performed using the SPAdes assembler with the setting ‘full assembly’ built in on the Pathosystems Resource Integration Center (PATRIC) server [14]. Gene prediction and annotation were performed using rapid annotation on the Subsystem Technology (RAST) server.

Next-generation sequencing (NGS) protocols generally yield sequencing of every base in the sample many times, depending on the depth of sequencing. The coverage metric is used to express the number of times the genome is sequenced in a given NGS experiment. Estimated sequence coverage of the genome in this study was obtained by the Lander–Waterman equation, *C*=*L·N*/*G*, where *C* stands for estimated coverage of the genome sequencing protocol, *G* is the expected genome length (using a closely relative genome as reference), *L* is the average size of reads (161 bp) and *N* is the total number of reads (16 606 977).

All genomes used were downloaded via file transfer protocol (FTP) from he RefSeq GenBank database. Sequences were aligned by Multiple Sequence Comparison by Log Expectation (muscle) [15] (options: -maxiters 1 -diags1) and trimmed using GBLOCK [16]. Variable aligned positions were removed by extracting single-nucleotide polymorphisms (SNPs) using SNP-SITES [17] and a maximum-likelihood phylogenetic tree was built using RaxML 8.2.9, with the ascertainment bias (ASC) parameter for variable sites and the resampling estimated log-likelihood (RELL) bootstrap technique for node support assessment.

The sequencing data from this study have been submitted to the National Center for Biotechnology Information’s (NCBI’s) BioProject (http://www.ncbi.nlm.nih.gov/bioproject) under BioProject accessions: SAMN10574394/VHIR00000000.

## Results and Discussion

**Fig. 1.**
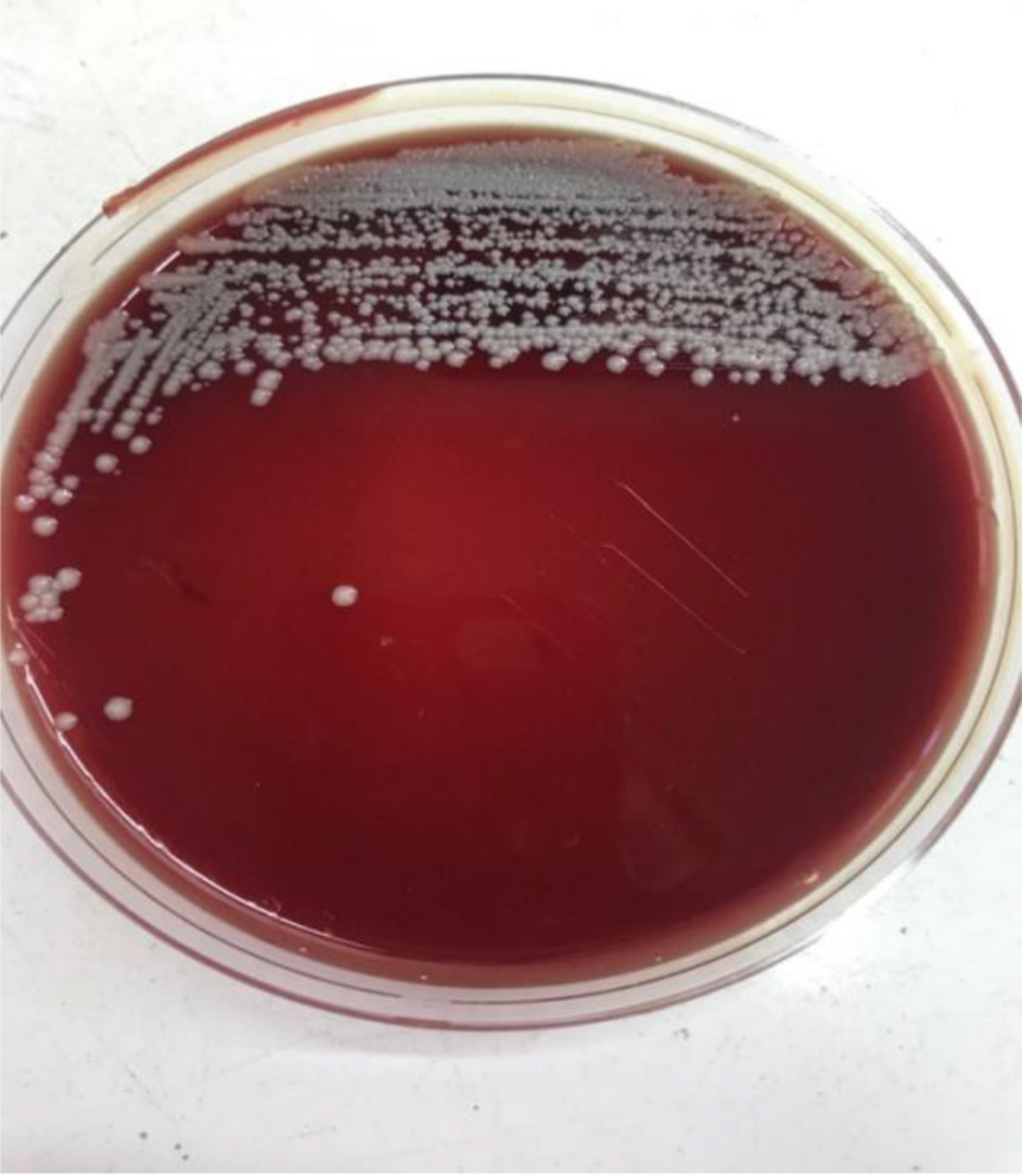
Morphology of *C*. *phoceense* colonies isolated in sheep blood agar, obtained from a urine sample. The colonies are whitish with no *beta* haemolysis.

Urine sediment microscopy showed leukocyturia [8–10 leukocytes per high-power field (HPF)] [18]. The culture showed a significant pure bacterial growth (>10^5^ c.f.u. ml^−1^) of whitish, non-haemolytic convex colonies with regular edges −1–1.5 mm in diameter after 24 h of incubation (Fig. 1).

The isolate consisted of Gram-positive, nonmotile, short rod-shaped cells that exhibited a coryneform morphology.

The organism was a catalase-positive, non-lipophilic, fermentative corynebacteria showing facultatively anaerobic growth. Positive results for aesculin, nitrate, pyrrolidonyl arylamidase, acid from glucose, maltose, sucrose and weak reaction for ribose were observed. The results obtained for β-glucuronidase, acid from lactose and CAMP reaction were negative.

The differential phenotypic characteristics of our strain (* Corynebacterium* sp. 272) were compared with those of other related non-liphophilic *Corynebacterium* species (Table 1).

**Table 1.** Differential phenotypic tests for related non-liphopilic *Corynebacterium* species isolated from a urine sample

Tests	Our strain	C. phoceense*	* C. striatum *	* C. amycolatum *	* C. aurimucosum *	* C. glucuronolyticum *
Esculin ^a/b^	+	nd	−	−	v	v
Nitrate ^a/b^	+	+	+/−	V	−	v
PYR ^a/b^	+	+	−	−	−	−
CAMP test ^b^	−	nd	v	−	nd	+
β glucuronidase ^a^	−	nd	−	−	−	+
Acid from: ^a^						
Glucose	+	+	+	+	+	+
Maltose	+	+	−	v	+	v
Sucrose	+	−	v	v	+	+
Ribose	+^w^	+	−	−	−	−
Lactose	−	+	−	−	−	−

Test results obtained by: ^a^ API Coryne, ^b^ Conventional biochemical tests. No discrepancies were observed between both tests.

*Results for the only strain described by Cresci *et al.*

^w^, weak reaction; PYR, pyrrolidonilarylamidase; nd, not determined.

The results for the phenotypic identification of our isolate showed similarities with those recently described by Cresci *et al.*, except for lactose and sucrose fermentation [9]. Since there are only published data for one strain of this species, we may conclude that the results of these tests might be variable for this species. Other species such as *C. aurimucosum* and *C. striatum* also share similar phenotypic characteristics (Table 1).

Based on the results of biochemical tests, aesculin hydrolysis, nitrate reduction, PYR activity and the absence of β glucuronidase activity were the most significant tests to differentiate *C. phoceense* from other non-lipophilic species with similar phenotypic characteristics that may be isolated from a urine sample (Table 1).

The isolation of a pure culture of coryneform bacteria indicates a definite criterion in terms of clinical significance [19]. This event associated with the patient’s underlying disease and symptoms indicates that this isolate could certainly be the cause of the UTI. This is the second description of a UTI involving *C. phoceense*. *C. phoceense* was recently isolated from the urine of a kidney transplant recipient. No other infectious diseases have been reported as being associated with this species.

Minimum inhibitory concentration (MIC) results showed susceptibility to penicillin (0.388 µg ml^−1^), ceftriaxone (0.5 µg ml^−1^), trimethoprim/sulfamethoxazole (TMS) (0.5 µg ml^−1^) and vancomycin (0.25 µg ml^−1^), and resistance to ciprofloxacin (8 µg ml^−1^). The patient was diagnosed as having a complicated UTI. She had been empirically treated with ciprofloxacin, but based on the antimicrobial susceptibility test, the antibiotic was rotated to TMS 160/800 mg every 12 h for 10 days with a favourable clinical outcome.

The strain was also identified using the MALDI Biotyper 3.1 (Bruker, Daltonics) as *Corynebacterium* sp. strain 901 400 365 LBK/901600604LBK (first and second top 10: 2.355/2.242, respectively) according to the MALDI TOF MS database 3.1, which contains 8468 MSP. *C. phoceense* was published in 2016 (https://pubmed.ncbi.nlm.nih.gov/27766158/), but as described elsewhere [9], it has not been validated yet. Based on the data provided by Bruker, the sequencing of the 16S rRNA gene of the *Corynebacterium* sp. strain 901 400 365 LBK (data not shown), as deposited in the database, matches *C. phoceense MC1* (GenBank LN 849777.1) and our strain (GenBank VHIR00000000) (99. 92 % nucleotide identity).

**Fig. 2.**
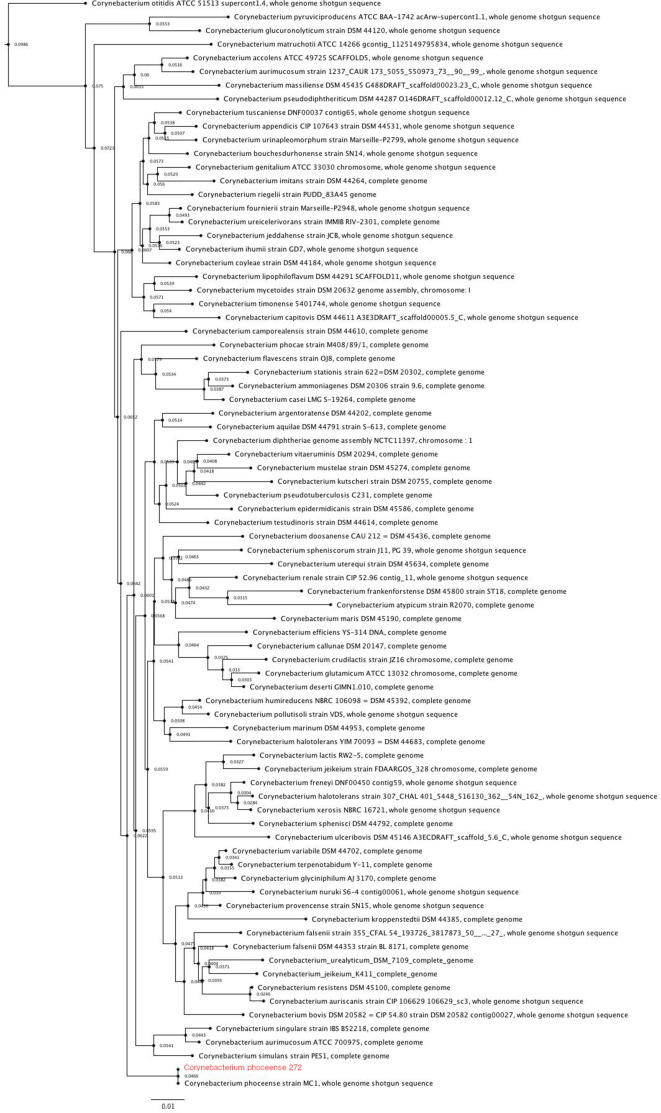
Maximum-likelihood phylogenetic tree of the 16S rRNA sequence of *Corynebacterium* species. Collection sequences were obtained from RefSeq, selecting representative genomes deposited at GenBank. The molecular evolution model used was the generalized time-reversible (GTR) model.

In order to determine the taxonomy group of our strain (* Corynebacterium* sp. 272), we performed 16S rRNA gene sequence analysis using Basic Local Alignment Search Tool (blast) software and the GenBank database. The best match against the GenBank database was *Corynebacterium* sp. NML 96–022 and *C. phoceense* MC1 with 100 % nucleotide identity. In order to understand the phylogeny of our strain, we performed a phylogenetic analysis of the 16S rRNA gene sequence, comparing it to 79 *Corynebacterium* genomes from RefSeq representative genomes of the GenBank database. The most phylogenetically related species to our strain was *C. phoceense* strain MC1, with 100 % identity. When we analysed *C. phoceense* as a taxonomic group, the most phylogenetically related species were *C. aurimucosum* strain ATCC 700975 and *Corynebacterium phocae* strain M408/89/1, with nucleotide identity of 96 % (Fig. 2).

**Fig. 3.**
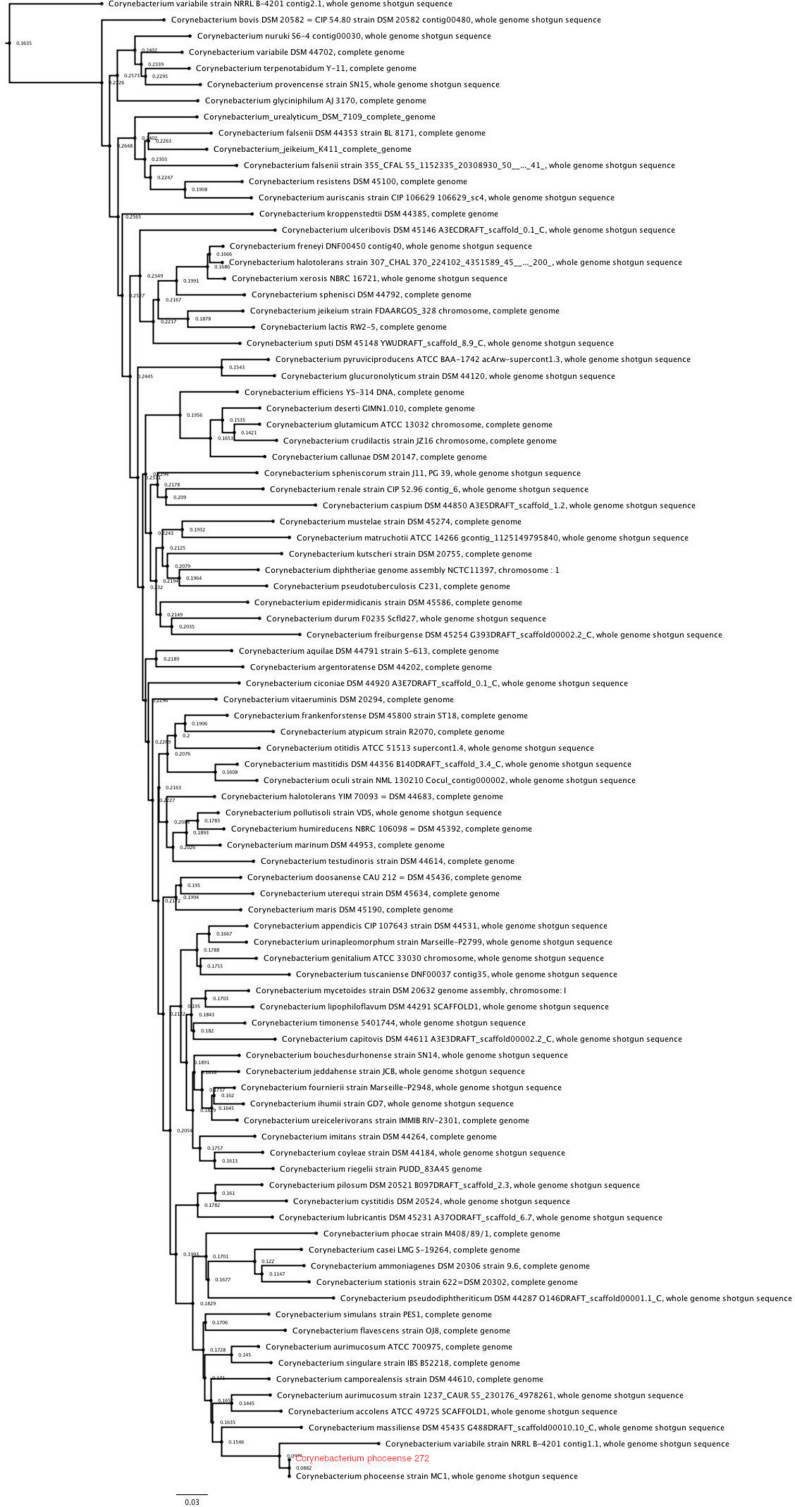
Maximum-likelihood phylogenetic tree of the β subunit of the RNA polymerase (*rpo*B*)* gene sequencing of *Corynebacterium* species. Collection sequences were obtained from RefSeq, selecting representative genomes deposited at GenBank. The molecular evolution model used was the generalized time-reversible (GTR) model.

In order to determine correct identification of the species, we performed a phylogenetic analysis of the β subunit of the RNA polymerase (*rpo*B) gene of our strain and of 100 *Corynebacterium* genomes from RefSeq representative genomes from the GenBank database [20]. In our phylogenetic analysis, we observed that the most related species to our strain was *C. phoceense* strain MC1, with a nucleotide identity of 99.9 %. When we analysed *C. phoceense* as a taxonomic group, the most related species was *C. aurimucosum* strain ATCC 700975, with a nucleotide identity of 97 % (Fig. 3).

Species identification of our strain was performed using the draft genome sequence and a custom database containing 144 complete or draft genomic sequences of *Corynebacterium* spp. Average nucleotide identity by blast+ and TETRA was calculated using JSpeciesWS [21]. The top hits obtained were against *C. phoceense* DSM 100570^T^ (ANIb=99.23 %/TETRA=0.99958). Values above the 95.0 % cutoff for ANIb and above the 0.99 cutoff for TETRA are considered to be the same species [22].

Digital DNA-to-DNA hybridization (dDDH) was calculated by GGDC 2.1, using formula 2, and rendered a DDH estimate of 94.30 % against *C. phoceense* DSM 100570^T^ (probability of DDH >70 %=97.04 %). Values above the 70.0 % cutoff are considered to be the same species [22].

A total of 16 606 977 high-quality reads were submitted to *de novo* assembly through the PATRIC server [14] and rendered an assembled genome with 96 contigs and an estimated genome size of 2 723 750 bp. The draft genome had an N50 contig size of 132 590 bp with a maximum contig length of 284 566 bp and a G+C content of 63.4 %. A total of 2698 protein-encoding genes (PEGs) and 52 tRNA genes and 7 rRNA genes were predicted using the PATRIC annotation server [14]. The annotation included 972 hypothetical proteins and 1726 proteins with functional assignments, of which 705 had Enzyme Commission (EC) number assignments and 603 had gene ontology (GO) assignments. The most commonly represented classes of genes were those involved in metabolism (490 genes), while 62 genes were assigned to the category of stress response, defence and virulence.

Whole-genome sequencing for the identification of the biochemical features of our isolate was performed using the strategy described by Santos *et al.* [23]. Of note, some biochemical reactions are shared by our isolate (*C. phoceense* 272) and the strain type of *C.phoceense* (MC1), while they are absent in *C.aurimucosum* ATCC700975 (e.g. EC 1.4.7.1 glutamate synthase and EC 1.7.99.4 nitrate reductase).

In summary, *C. phoceense* has just been described in two events related to UTI in patients with underlying diseases. Recently, species belonging to the genus *Corynebacterium* have been considered to be emerging pathogens involved in UTIs. With this description, we would like to highlight the importance of recognizing new species as potential pathogens for UTIs, especially in patients with predisposing factors.

## Conclusion

Based on phylogenetic and genomic analyses, we assume that the strain could be identified as the second isolate of *Corynebacterium phoceense* to be described. Since both isolates were isolated from a urine sample, we suggest that the relationship of this new species with urinary tract infections should be taken into account.
